# Classification and Morphological Parameters of the Calcaneal Talar Facet: Which Type Is More Likely to Cause Osteoarthritis in Chinese Population?

**DOI:** 10.1155/2019/6095315

**Published:** 2019-04-04

**Authors:** Yi Yang, Han-wen Cheng, Zheng-rong Xiong, Nian Liu, Yang Liu, Yi Wang, Yan Zeng, Shi-jie Fu, Lei Zhang

**Affiliations:** ^1^Department of Obstetrics, Chengdu Women's and Children's Central Hospital, No. 1617 of Section One, Riyue Ave. Qingyang District, Chengdu, Sichuan Province, China; ^2^School of Clinical Medicine, Southwest Medical University, Luzhou, China; ^3^Academician Workstation Construction Project of Luzhou, Sichuan Province, China; ^4^Department of Orthopedics, Affiliated Traditional Chinese Medicine Hospital of Southwest Medical University, Luzhou, China; ^5^School of Traditional Chinese and Western Medicine, Southwest Medical University, Luzhou, China; ^6^Department of Nephrosis Internal Medicine, Affiliated Traditional Chinese Medicine Hospital of Southwest Medical University, Luzhou, China; ^7^National Key Discipline of Human Anatomy, School of Basic Medical Sciences, Southern Medical University, Guangzhou, China

## Abstract

Due to the calcaneal osteoarthritis, patients had a lower quality of life. This research was to study which type of calcaneus was more likely to cause osteoarthritis and then to guide the clinical prevention and treatment in Chinese population. All 505 intact Chinese calcaneus facets were reconstructed by CT-3D reconstruction scanner and classified into five types based on the calcaneal talar facet (CTF) configuration. CTF's morphology parameters (osteophyte, cortical thickness of calcaneus, GIssane's and Bohler's angle, and long and short axis) were measured and recorded by PACS CT system. Researchers used the length of long and short axis to calculate the CTF area. By comparing the morphology parameters of five types of calcaneus, the differences among different types of calcaneus in Chinese people were statistically different. The study showed that Type II and Type IV had the highest percentage of osteophytes. After being compared and analyzed, the CTF pressure and the subtalar joint stability were closely related to the occurrence of osteoarthritis. Based on the measurement and comparison of morphological parameters in this study, Types II and IV were the most likely to develop osteoarthritis in Chinese population.

## 1. Introduction

The calcaneal and talus were connected through the articular surface, which stabilized and supported the subtalar joint. Furthermore the calcaneal talar facet (CTF) was a characteristic structure of the calcaneus. In general, the CTF was divided into three parts. However, the reality was that there was a variation in the morphology of the CTF [1–6]. Some of these variants were more likely to cause joint degeneration and even osteoarthritis [2, 7]. With the increase of osteoarthritis, the knowledge of calcaneus' classification and morphological parameters was critically important for calcaneal osteoarthritis. This research was focused on the relationship between several different types of CTF and calcaneal osteoarthritis in Chinese population.

Research on the morphology of CTF had a long tradition [1–3, 8–10]. There were several significant types in Europe, Africa, and other continents. Madhavi [2] had found five types of calcaneal talar facet in south Indian, and Uygur M [3] had defined some types of CTF in Turkey. The osteophyte on the CTF was a key factor in the formation of osteoarthritis [11]; the formation of osteophyte was associated with several specific types of CTF [2] and patients' age [12–15]. Besides, Krähenbühl [7] had found that the specific morphology of CTF would result in an asymmetric wear pattern which straightly leads to osteoarthritis. The reason for this phenomenon was that the patients' abnormal CTF would cause heavy pain. The subtalar and tibiotalar joint were habitually inclined when patients walked [16, 17]. With time going by, patients' subtalar joint would be damaged and CTF could form osteophytes that eventually resulted in osteoarthritis [7, 11, 17, 18]. Several similar kinds of animal experiments had been widely accepted. The hominoid primates' morphology of the CTF was associated with the compression of the joints; particularly, the types of CTF with insufficient compression were susceptible to osteoarthritis [4]. In clinical treatment, more detailed knowledge of CTF was helpful for orthopedic surgery and osteoarthritis patients.

However, in recent decades, the researches on classification and measurement of morphological parameters were not comprehensive. More and more osteoarthritis cases were caused by calcaneus fracture or long-term wear [17, 19–22]. Doctors are reluctant to perform surgery on the calcaneus because of the uncertainty and the high incidence of complications, although operative treatment was better than nonoperative treatment in terms of prognosis and economics [23–28]. Comprehensive and detailed classification can better assist doctors in the surgery and eliminate secondary complications [12]. The key contribution of this research was the solution it provided, a more detailed morphological parameters of Chinese CTF, and the study of the variants' effects on osteoarthritis which were rarely recorded in previous literature. This research could also make people know Chinese calcaneal anatomy better and assist arthroscopic reconstruction of the CTF in calcaneal fractures to prevent osteoarthritis.

## 2. Materials and Methods

### 2.1. Ethical Statement

Ethical approval was given by the Medical Ethics Committee of Southwest Medical University with the following reference number: KY2018012.

### 2.2. Materials

A total of 505 intact Chinese calcaneus facets were collected from the Affiliated Traditional Chinese Medicine Hospital of Southwest Medical University, including Chinese calcaneus with varying degrees of CTF degeneration. Patients with congenital calcaneal deformities and calcaneal fractures were excluded. There were 293 left calcaneus cases (176 male, 116 female) with patients' average age of 41 years (range, 18-80) and 212 right calcaneus cases (132 male, 81 female) with average age of 42 years (range, 18-80). All calcaneus facets reconstructed by CT-3D reconstruction scanner had no deformities and no fractures.

### 2.3. Methods for Classification of CTF

**Figure 1:**
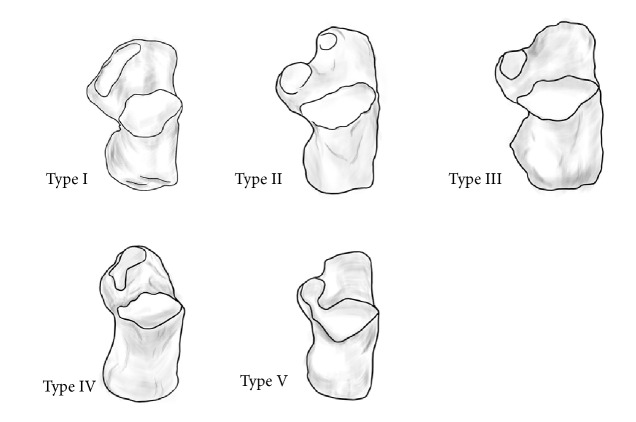
*The morphology of five types of calcaneal talar facet*. Type I: anterior and middle facets were fused into a pear-shaped big facet. Type II: anterior, middle, and posterior three separate facets. Type III: missing anterior facet. Type IV: anterior and middle calcaneus facets were fused into a calabash-shaped big facet. Type V: absence of the anterior facet, and the middle and posterior ones were fused.

A CT three-dimensional reconstruction scanner was used to reconstruct and capture the facets morphology of calcaneus. The classification was based on the shape, quantity, and fusion of the joint surface which was observed by investigators. Calcaneus classification was carried out simultaneously by two medical practitioners who had worked for more than 10 years. If there were any differences in the results, the third medical practitioner would be responsible for the judgment. Five specific types of CTF were noted: Type I, anterior and middle calcaneus facets were fused into a pear-shaped facet, and the posterior facet was separated; Type II, anterior, middle, and posterior facets were all separated; Type III, absence of the anterior facet, the middle and posterior facet were separated; Type IV, anterior and middle calcaneus facets were fused into a calabash-shaped facet, and the posterior facet was separated; Type V, absence of the anterior facet, the middle and posterior facet were partially connected and fused into one facet (Figure 1).

### 2.4. Methods for Measuring the Morphological Parameters

The different types of calcaneal talar facet were recorded in number and percentage. The GIssane's and Bohler's angles were measured by a protractor (Model 6002, provided by Zhejiang Chute Co., Ltd.).

Average total joint facet area was calculated from the measured parameters. Each individual joint surface was approximately a rectangle, and the area was calculated by using the values of the long and short axes. The long and short axis were carried out and recorded by vernier caliper (Model HXC-0-150MM, accurate to 0.1 mm, provided by Zhejiang Jiechao Machinery Factory). In order to avoid errors between the observers, all the calcaneal bones were carefully measured and recorded by 2 investigators. The investigators were medical practitioners, who had worked for more than ten years, from the Affiliated Traditional Chinese Medicine Hospital of Southwest Medical University.

### 2.5. Statistical Methods

All measurements were expressed as mean and standard deviation (SD). Categorical variables were described by number and percentages. The homogeneity of variance was performed by using Shapiro-Wilk test. One-way ANOVA was used to compare 5 types of CTF, considering a P-value < 0.05 as statistically significant. IBM SPSS Statistics, version 17.0 (Chicago, IL), was used for all statistical analyses.

## 3. Results

**Figure 2:**
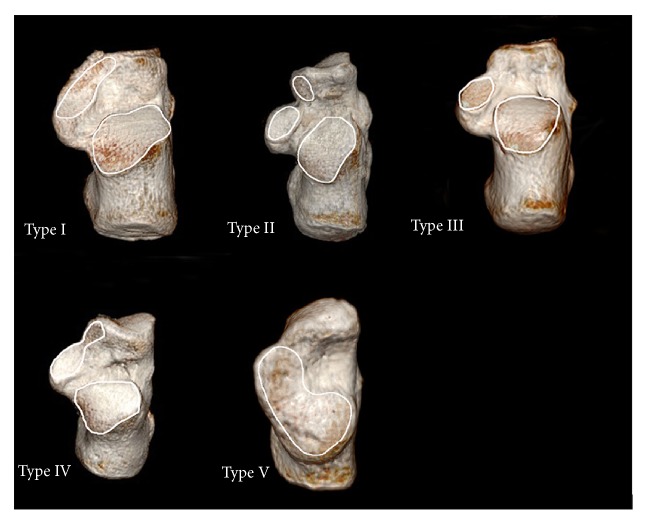
*Three-dimensional CT reconstruction of calcaneal talar facet*. Type I: anterior and middle facets were fused into a pear-shaped big facet. Type II: three separate anterior, middle, and posterior facets. Type III: missing anterior facet. Type IV: anterior and middle calcaneus facets were fused into a calabash-shaped big facet. Type V: absence of the anterior facet, and the middle and posterior ones were fused.

The Chinese calcaneus was classified into five types based on the morphology of CTF (Figures 1 and 2).

Five specific types of CTF were noted: Type I, anterior and middle calcaneus facets were fused into a pear-shaped facet, and the posterior facet was separated; Type II, anterior, middle, and posterior facets were all separated; Type III, absence of the anterior facet, the middle and posterior facet were separated; Type IV, anterior and middle calcaneus facets were fused into a calabash-shaped facet, and the posterior facet was separated; Type V, absence of the anterior facet, the middle and posterior facet were partially connected and fused into one facet.

According to Table 1, 140 osteophytes were noticed in 255 Type I calcaneus facets (54.9%), 66 were noticed in 113 Type II calcaneus facets (58.4%), 36 were noticed in 76 Type III calcaneus facets (47.4%), 31 were noticed in 52 Type IV calcaneus facets (59.6%), and 2 were noticed in 9 Type V calcaneus facets (22.2%).

The average cortical thickness of Type III (4.31±1.53 mm) was thinner than Type II (4.98±1.88 mm) (p<0.05); Type IV (5.19±1.23 mm) was thicker than Type I (4.67±1.64 mm) and Type III (4.31±1.53 mm) (p<0.05).

Table 2 showed the average sum of joint facet area: Type III (7.07±1.44 cm^2^) was significantly smaller than Type I (8.38±1.76 cm^2^), II (8.13±1.64 cm^2^), and V (9.31±3.96 cm^2^) (p<0.05). Besides, Type IV (7.71±1.68 cm^2^) was smaller than Type I (8.38±1.76 cm^2^) and V (9.31±3.96 cm^2^); the statistical difference between them was significant (p<0.05).

In the mean GIssane's angle, there was no statistical difference among the five types of calcaneus (p<0.05).

The mean Bohler's angle of Type II (28.96±6.82°) was smaller than Type I (30.79±4.86°), III (30.63±5.21°), and IV (31.22±4.30°), and the difference was statistically significant (p<0.05).

## 4. Discussion

Calcaneal osteoarthritis was a serious disease that greatly reduced the quality of life and even led to disability [16, 17, 19]. Studies have shown that abnormal wear and increased joint load can lead to tissue damage, damage the ability of chondrocytes to maintain and restore tissue, and play an important role in the development of joint degeneration [6, 12, 16, 18]. Madhavi [2] has demonstrated that articular surface configuration and various morphological parameters were related to the occurrence of osteoarthritis in south Indian populations [23].

In this research, based on the unique shape of each calcaneus, all 505 calcaneus facets were carefully classified into five types by investigators. The result showed that Type I was the most common in Chinese population, followed by Type II, III, and IV. There were only nine calcaneus facets under Type V. According to the mean calcaneal cortical thickness of the five types, Type IV (5.19±1.23 mm) was obviously thicker than Type I (4.67±1.64 mm) and Type III (4.31±1.53) mm. Type II (4.98±1.88 mm) was thicker than Type III (4.31±1.53 mm) (p<0.05). It turned out that Type II and IV had more wear and tear, leading to more severe joint degeneration. This phenomenon may be caused by excessive pressure on the surface of the joint, leading directly to the thickening of the bone cortex on the surface of the joint.

Under the same load, one of the key factors affecting joint surface pressure was the size of joint surface area, the greater pressure more easily damaged the tissue of the CTF [12, 18, 29]. Type IV (7.71±1.68 cm^2^) was obviously smaller than Type I (8.38±1.76 cm^2^) and Type V (9.31±3.96 cm^2^); the difference was statistically significant (p<0.05). It illustrated that the joint surface pressure in Type IV was greater than the other two types, and Type IV was more likely to cause joint surface degeneration, which was consistent with the percentage of osteophyte (Table 1). Type III (7.07±1.44 cm^2^) was smaller than Type V (9.31±3.96 cm^2^) (p<0.05), and the percentage of osteophyte also indicated that Type III was more than Type V (Table 1).

Another risk factor that affected joint surface pressure was the Bohler's angle. When the Bohler's angle decreased, the joint surface pressure would increase, because the contact characteristics of the joint surface changed [30]. Type II (28.96±6.82°) was sharper than Type I (30.79±4.86°), Type III (30.63±5.21°), and Type IV (31.22±4.30°). The difference was statistically significant (p<0.05). It means the joint surface pressure of Type II was greater than Type I and Type III; this would make Type II develop joint degeneration more easily.

Regarding the GIssane's angle in this study, the five types of GIssane's angle were similar (Table 2), but there was no statistical difference among the five calcaneal types (p>0.05). This might be due to the small sample size.

Benjamin [11] has found that osteophytes formed in the articular cartilage when osteoarthritis occurred, and in this study, the percentage of osteophyte showed that Type IV (59.6%) and Type II (58.4%) were obviously more than Type I (54.9%), Type III (47.4%), and Type V (22.2%), which indicated that Type IV and Type II were the most likely to develop osteoarthritis in five types of Chinese calcaneus facets.

However, limitations still exist in this study. The morphological parameters of the calcaneus samples in this experiment may have errors due to CT three-dimensional reconstruction, and this study explored the differences between different types of calcaneus facets, without exploring the differences between sexes.

## 5. Conclusions

By comparison and analysis, this study indicated that Type II and IV were more likely to cause osteoarthritis in Chinese population. The occurrence of osteoarthritis was closely related to the value of Bohler's angle and the area of joint surface which affected the calcaneal talar facet pressure and the subtalar joint stability, respectively.

## Figures and Tables

**Table 1:** Comparison of the osteophyte and the cortical thickness of calcaneus in different types of calcaneus ($\overset{-}{x}$±s).

	No.	Percentage of calcaneus	Percentage of osteophyte	Average cortical thickness of calcaneus (mm)
Type I	255	50.5%	54.9%	4.67±1.64
Type II	113	22.4%	58.4%	4.98±1.88
Type III	76	15.0%	47.4%	4.31±1.53^b^
Type IV	52	10.3%	59.6%	5.19±1.23^ac^
Type V	9	1.8%	22.2%	4.68±1.54

^a^ p<0.05 vs Type I, ^b^ p<0.05 vs Type II, ^c^ p<0.05 vs Type III.

**Table 2:** Measurements of calcaneus morphological parameters based on classification ($\overset{-}{x}$±s).

	Average total joint facet area (cm^2^)	Mean GIssane's angle (°)	Mean Bohler's angle (°)
Type I	8.38±1.76	131.4±7.24	30.79±4.86
Type II	8.13±1.64	130.5±7.64	28.96±6.82^a^
Type III	7.07±1.44^ab^	130.5±6.28	30.63±5.21^b^
Type IV	7.71±1.68^a^	132.7±7.24	31.22±4.30^b^
Type V	9.31±3.96^cd^	131.4±3.46	31.26±6.31

^a^ p<0.05 vs Type I, ^b^ p<0.05 vs Type II, ^c^ p<0.05 vs Type III, ^d^ p<0.05 Type IV.

## Data Availability

The related calcaneal talar facet data used to support the findings of this study are restricted by the Ethical Inspection Committee at Southwest Medical University (No. KY2018012). Data are available from Lei Zhang (email: zhanglei870722@126.com) for researchers who meet the criteria for access to confidential data.

## References

[1] Gupta S. C., Gupta C. D., Arora A. K. (1977). Pattern of talar articular facets in Indian calcanei. *Journal of Anatomy*.

[2] Madhavi C., Madhuri V., George V. M., Antonisamy B. (2008). South Indian calcaneal talar facet configurations and osteoarthritic changes. *Clinical Anatomy*.

[3] Uygur M., Atamaz F., Celik S., Pinar Y. (2009). The types of talar articular facets and morphometric measurements of the human calcaneus bone on Turkish race. *Archives of Orthopaedic and Trauma Surgery*.

[4] Parr W. C., Soligo C., Smaers J. (2014). Three-dimensional shape variation of talar surface morphology in hominoid primates. *Journal of Anatomy*.

[5] Ragab A., Stewart S., Cooperman D. Implications of subtalar joint anatomic variation in calcaneal lengthening osteotomy. *Journal of Pediatric Orthopaedics*.

[6] Krähenbühl N., Tschuck M., Bolliger L., Hintermann B., Knupp M. (2016). Orientation of the subtalar joint:measurement and reliability using weightbearing CT scans. *Foot & Ankle International*.

[7] Bunning P. S. (1965). A comparison of adult and foetal talocalcaneal articulations. *Journal of Anatomy*.

[8] Acer N., Bayar B., Basaloglu H., Öner E., Bayar K., Sankur S. (2008). Unbiased estimation of the calcaneus volume using the Cavalieri principle on computed tomography images. *Annals of Anatomy*.

[9] Yapuncich G. S., Boyer D. M. (2014). Interspecific scaling patterns of talar articular surfaces within primates and their closest living relatives. *Journal of Anatomy*.

[10] Benjamin M., Toumi H., Suzuki D., Hayashi K., McGonagle D. (2009). Evidence for a distinctive pattern of bone formation in enthesophytes. *Annals of the Rheumatic Diseases*.

[11] Buckwalter J. A., Martin J. A. (2006). Osteoarthritis. *Advanced Drug Delivery Reviews*.

[12] Lawrence R. C., Hochberg M. C., Kelsey J. L., etak (1989). Estimates of the prevalence of selected arthritic and musculoskeletal diseases in the United States. *The Journal of Rheumatology*.

[13] Lawrence J. S., Bremner J. M., Bier F. (1966). Osteo-Arthrosis: Prevalence in the Population and Relationship between Symptoms and *X* -ray Changes. *Annals of the Rheumatic Diseases*.

[14] van Saase J. L., van Romunde L. K., Cats A., Vandenbroucke J. P., Valkenburg H. A. (1989). Epidemiology of osteoarthritis: Zoetermeer survey. Comparison of radiological osteoarthritis in a Dutch population with that in 10 other populations. *Annals of the Rheumatic Diseases*.

[15] Cooper C., Adachi J. D., Bardin T. (2013). How to define responders in osteoarthritis. *Current Medical Research and Opinion*.

[16] Wang B., Saltzman C. L., Chalayon O., Barg A. (2015). Does the subtalar joint compensate for ankle malalignment in end-stage ankle arthritis?. *Clinical Orthopaedics and Related Research*.

[17] Li J., Muehleman C. (2007). Anatomic relationship of heel spur to surrounding soft tissues: Greater variability than previously reported. *Clinical Anatomy*.

[18] Barg A., Pagenstert G. I., Hügle T. (2013). Ankle osteoarthritis: etiology, diagnostics, and classification. *Foot and Ankle Clinics*.

[19] Franke J., Wendl K., Suda A. J., Giese T., Grützner P. A., von Recum J. (2014). Intraoperative three-dimensional imaging in the treatment of calcaneal fractures. *The Journal of Bone and Joint Surgery-American Volume*.

[20] Chang C. B., Jeong J. H., Chang M. J., Yoon C., Song M. K., Kang S. (2018). Concomitant ankle osteoarthritis is related to increased ankle pain and a worse clinical outcome following total knee arthroplasty. *The Journal of Bone & Joint Surgery*.

[21] van der Vliet Q. M. J., Hietbrink F., Casari F., Leenen L. P. H., Heng M. (2018). Factors influencing functional outcomes of subtalar fusion for posttraumatic arthritis after calcaneal fracture. *Foot & Ankle International*.

[22] Brauer C. A., Manns B. J., Ko M., Donaldson C., Buckley R. (2005). An economic evaluation of operative compared with nonoperative management of displaced intra-articular calcaneal fractures. *The Journal of Bone & Joint Surgery*.

[23] Petit C. J., Lee B. M., Kasser J. R., Kocher M. S. (2007). Operative treatment of intraarticular calcaneal fractures in the pediatric population. *Journal of Pediatric Orthopaedics*.

[24] Gamie Z., Donnelly L. (2009). The "safe zone" in medial percutaneous calcaneal pin placement. *Clin Anat*.

[25] Griffin D., Parsons N., Shaw E. (2014). Operative versus non-operative treatment for closed, displaced, intra-articular fractures of the calcaneus: Randomised controlled trial. *BMJ*.

[26] Tantavisut S., Phisitkul P., Westerlind B. O., Gao Y., Karam M. D., Marsh J. L. (2016). Percutaneous reduction and screw fixation of displaced intra-articular fractures of the calcaneus. *Foot & Ankle International*.

[27] Bruce J. (2013). Surgical versus conservative interventions for displaced intra-articular calcaneal fractures. *The Cochrane database of systematic reviews*.

[28] Chen C.-H., Hung C., Hsu Y.-C., Chen C.-S., Chiang C.-C. (2017). Biomechanical evaluation of reconstruction plates with locking, nonlocking, and hybrid screws configurations in calcaneal fracture: a finite element model study. *Medical & Biological Engineering & Computing*.

[29] Probasco W., Haleem A. M., Yu J., Sangeorzan B. J., Deland J. T., Ellis S. J. (2015). Assessment of coronal plane subtalar joint alignment in peritalar subluxation via weight-bearing multiplanar imaging. *Foot & Ankle International*.

[30] Jones C. R., Wong E., Applegate G. R., Ferkel R. D. (2018). Arthroscopic ankle arthrodesis: A 2-15 year follow-up study. *Arthroscopy - Journal of Arthroscopic and Related Surgery*.

